# Identification of a haplotype block in the 5q31 cytokine gene cluster associated with the susceptibility to severe malaria

**DOI:** 10.1186/1475-2875-8-232

**Published:** 2009-10-19

**Authors:** Izumi Naka, Nao Nishida, Jintana Patarapotikul, Pornlada Nuchnoi, Katsushi Tokunaga, Hathairad Hananantachai, Naoyuki Tsuchiya, Jun Ohashi

**Affiliations:** 1 Doctoral Programme in Life System Medical Sciences, Graduate School of Comprehensive Human Sciences, University of Tsukuba, Ibaraki, Japan; 2 Department of Human Genetics, Graduate School of Medicine, The University of Tokyo, Tokyo, Japan; 3 Faculty of Tropical Medicine, Mahidol University, Bangkok, Thailand; 4 Department of Clinical Microscopy, Faculty of Medical Technology, Mahidol University, Bangkok, Thailand

## Abstract

**Background:**

It has been previously demonstrated that a single nucleotide polymorphism (SNP) in the *IL13 *promoter region, *IL13 *-1055T>C (rs1800925), was associated with susceptibility to severe malaria in Thais. In the present study, fine association mapping for a cytokine gene cluster including *IL4*, *IL5*, and *IL13 *on chromosome 5q31 was conducted using the same malaria subjects to refine the region containing a primary variant or a haplotype susceptible to severe malaria.

**Methods:**

A total of 82 SNPs spanning 522 kb of the 5q31 region were analysed in 368 patients with *Plasmodium falciparum *malaria (203 mild malaria and 165 severe malaria patients).

**Results:**

Only rs1881457 located in the promoter region of *IL13*, which is in linkage disequilibrium with rs1800925 (r^2 ^= 0.73), showed a significant association with severe malaria after adjusting for multiple testing (P = 0.046 by permutation test). This SNP was in a haplotype block spanning 97 kb (from rs2069812 to rs2240032). The detected haplotype block contained the *RAD50 *gene and the promoter of *IL13*, but not the other genes.

**Conclusion:**

A haplotype block in which a primary polymorphism associated with severe malaria is likely to be encoded was identified in Thai malaria patients.

## Background

Over the course of the last decade a number of studies have provided evidence for a linkage between the blood infection level of *Plasmodium falciparum *and the human chromosome 5q31 region in African populations [1-4]. In addition to malaria, the 5q31 region shows a linkage to the response against other infectious diseases such as schistosomiasis [5] and leishmaniasis [6]. The 5q31-33 region contains genes encoding the T helper 2-type cytokines (the interleukin genes *IL3*, *IL4*, *IL5*, *IL9*, and *IL13*) and other immunologically active genes such as interferon regulatory factor-1 (*IRF1*). These genes are strong candidates for controlling the outcome of malaria infection.

In a previous study, three single nucleotide polymorphisms (SNPs) in the promoter regions of *IL3*, *IL4*, and *IL13 *were investigated. Of which, a SNP in the *IL13 *promoter region, *IL13 *-1055T>C (rs1800925), was found to be associated with susceptibility to severe malaria in Thais [7]. However, a number of candidate genes or polymorphisms still remain to be analyzed. In addition, no other polymorphisms surrounding rs1800925 were analyzed and thus the possibility that the association of rs1800925 with severe malaria may have resulted from linkage disequilibrium (LD) from other polymorphisms could not be excluded. The aim of this study is to better define the genomic region showing the association with severe malaria on the 5q31 region.

## Methods

### Subjects

A total of 368 adult patients with *P. falciparum *malaria (165 patients with severe *P. falciparum *malaria and 203 patients with mild malaria) living in northwest Thailand were enrolled in this study. All patients underwent treatment at the Hospital for Tropical Diseases, Faculty of Tropical Medicine, Mahidol University. Malarial infection by *P. falciparum *was confirmed by a positive blood smear for the asexual form of *P. falciparum*. Clinical manifestations of severe and mild malaria were classified according to the following definitions and criteria. A patient was classified as severe malaria when he/she has one or more of the following signs: high parasitaemia (>100,000 parasite/ml), hypoglycaemia (glucose <22 nmol/l), severe anaemia (haematocrit <20% or haemoglobin <7.0 g/dl), and a serum creatinine level of more than 3.0 mg/dl. In the present study, patients with cerebral malaria were not analyzed. Mild malaria was characterized by fever without other any underlying causes of infections and no manifestations of severe malaria as described above. All individuals were 13 years of age or older, and the mean ages of patients with mild malaria and those with severe malaria were 25.3 and 23.8, respectively. This study was approved by the institutional review board of the Faculty of Tropical Medicine, Mahidol University, and the Research Ethics Committees of the Faculty of Medicine, The University of Tokyo, and the Graduate School of Comprehensive Human Sciences, University of Tsukuba. Informed consent was obtained from all participants.

### Genotyping

Genomic DNA was extracted from peripheral blood leukocytes using a QIAamp Blood Kit (Qiagen, Hilden, Germany). A total of 82 SNPs within a 522 kb region on human chromosome 5q31 were genotyped by using the DigiTag2 assay [8] or TaqMan assay (Table 1). These SNPs were selected to capture the LD structure on 5q31 in Asian populations [9].

**Table 1:** Allele frequencies and association tests for SNPs in 5q31 cytokine cluster

**SNP rs#**	**Gene**	**Allelic state**		**Frequency of derived allele**		**Association P value**	
				
		**Ancestral**	**Derived**	**Severe malaria**	**Mild malaria**	**Raw**	**Permutation**
rs162887	SLC22A4	C	T	0.369	0.402	0.381	1.000
rs3792876	SLC22A4	C	T	0.234	0.236	0.944	1.000
rs3792878	SLC22A4	G	A	0.954	0.932	0.235	0.996
rs3805665	SLC22A4	G	A	0.23	0.237	0.823	1.000
rs3805668	SLC22A4	G	A	0.229	0.236	0.818	1.000
rs270608	SLC22A4	A	G	0.338	0.379	0.328	1.000
rs270607	SLC22A4	C	T	0.372	0.397	0.515	1.000
rs2073839	SLC22A4	C	T	0.234	0.236	0.950	1.000
rs3828673	SLC22A4	G	A	0.234	0.236	0.950	1.000
rs3792885	SLC22A4	A	T	0.229	0.236	0.818	1.000
rs272842	SLC22A4	T	C	0.348	0.299	0.181	0.985
rs3761659	SLC22A4	C	G	0.234	0.236	0.950	1.000
rs3805673	SLC22A4	G	A	0.228	0.237	0.771	1.000
rs273915	SLC22A4	G	C	0.375	0.397	0.563	1.000
rs272887	SLC22A4	C	T	0.372	0.397	0.515	1.000
rs273909	SLC22A4	T	C	0.071	0.093	0.307	1.000
rs272879	SLC22A4	G	C	0.344	0.297	0.201	0.992
rs272873	SLC22A4	C	T	0.167	0.175	0.773	1.000
rs2306772	SLC22A4	G	A	0.234	0.236	0.950	1.000
rs272867		C	T	0.344	0.298	0.205	0.992
rs3788987	SLC22A5	G	A	0.232	0.238	0.843	1.000
rs2631362	SLC22A5	T	C	0.365	0.376	0.769	1.000
rs2631359	SLC22A5	G	A	0.363	0.379	0.671	1.000
rs4646301	SLC22A5	G	A	0.236	0.253	0.614	1.000
rs274571	SLC22A5	T	C	0.365	0.379	0.719	1.000
rs2073642	SLC22A5	C	T	0.241	0.254	0.707	1.000
rs183898	SLC22A5	G	C	0.361	0.381	0.597	1.000
rs4646305	SLC22A5	G	A	0.237	0.251	0.678	1.000
rs274559	SLC22A5	C	T	0.348	0.299	0.181	0.985
rs274558	SLC22A5	C	T	0.353	0.299	0.144	0.965
rs274554	SLC22A5	A	G	0.837	0.887	0.059	0.748
rs274553	SLC22A5	G	C	0.163	0.113	0.059	0.748
rs274551	SLC22A5	C	T	0.161	0.113	0.072	0.804
rs274549	SLC22A5	G	T	0.163	0.113	0.059	0.748
rs274547	SLC22A5	T	A	0.839	0.887	0.072	0.804
rs2285673	LOC441108	C	T	0.227	0.199	0.388	1.000
rs2269822	LOC441108	C^b^	T	0.282	0.281	1.000	1.000
rs3749834		C	T	0.236	0.238	0.929	1.000
rs2070730	IRF1	C	T	0.418	0.407	0.765	1.000
rs2070727	IRF1	G	T	0.42	0.406	0.725	1.000
rs2070723	IRF1	C	T	0.582	0.593	0.765	1.000
rs2070722	IRF1	G	T	0.596	0.587	0.823	1.000
rs739718^a^		A	G	0.257	0.261	0.903	1.000
rs2069812^a^	IL5	T	C	0.241	0.302	0.083	0.848
rs2299015	RAD50	A	C	0.111	0.186	0.008	0.193
rs2299014	RAD50	T	G	0.074	0.07	0.838	1.000
rs2243677	RAD50	G	A	0.887	0.804	0.004	0.107
rs2522414	RAD50	G	C	0.887	0.804	0.004	0.107
rs2299013	RAD50	C^c^	G	0.104	0.186	0.004	0.101
rs2252775	RAD50	A	C	0.11	0.186	0.007	0.174
rs2522394	RAD50	A	G	0.887	0.804	0.004	0.107
rs2245460	RAD50	A	T	0.112	0.189	0.006	0.160
rs2301713	RAD50	T	C	0.113	0.186	0.010	0.242
rs3798135	RAD50	G	A	0.113	0.186	0.010	0.242
rs2237060	RAD50	A	C	0.04	0.055	0.351	1.000
rs2074369	RAD50	C	T	0.883	0.804	0.006	0.158
rs2240032	RAD50	C	T	0.106	0.183	0.006	0.152
rs1881457	IL13	A	C	0.124	0.219	0.002	0.046^d^
rs1800925	IL13	T	C	0.894	0.826	0.014	0.296
rs2066960	IL13	A	C	0.628	0.636	0.830	1.000
rs20541^a^	IL13	C	T	0.33	0.389	0.115	0.924
rs2070874	IL4	T	C	0.273	0.274	0.975	1.000
rs2243270	IL4	G	A	0.263	0.266	0.913	1.000
rs2243289	IL4	A	G	0.729	0.736	0.825	1.000
rs1468215	KIF3A	T	A	0.26	0.247	0.725	1.000
rs3798132	KIF3A	A	G	0.352	0.336	0.685	1.000
rs3798130	KIF3A	G	A	0.671	0.677	0.882	1.000
rs2299007	KIF3A	T	C	0.383	0.431	0.219	0.994
rs2237057	KIF3A	T	C	0.742	0.75	0.818	1.000
rs2299006	KIF3A	G	C	0.654	0.667	0.738	1.000
rs2299005	KIF3A	T	C	0.661	0.668	0.836	1.000
rs3798129	KIF3A	A	T	0.336	0.337	0.975	1.000
rs3756754	KIF3A	C	T	0.022	0.013	0.366	1.000
rs256871	SEPT8	C	T	0.609	0.635	0.490	1.000
rs30534	SEPT8	G	A	0.391	0.365	0.490	1.000
rs30533	SEPT8	T	C	0.386	0.371	0.686	1.000
rs39588	SEPT8	C	G	0.383	0.369	0.717	1.000
rs256875	SEPT8	T	C	0.385	0.379	0.888	1.000
rs392916	SEPT8	T	A	0.532	0.561	0.459	1.000
rs30527	SEPT8	C	T	0.62	0.639	0.620	1.000
rs30524	SEPT8	G	T	0.383	0.362	0.573	1.000
rs757537	ANKRD43	T	C	0.131	0.157	0.357	1.000

^a ^SNP genotyped by TaqMan assay.

^b ^Allelic state could not be inferred.

^c ^Allelic state inferred based on the sequence of rhesus macaque.

^d ^Permutation P value < 0.05.

### Statistical analysis

The allele frequency at each SNP locus was compared between mild and severe malaria patients using the chi-square test and a permutation P value was calculated from 100000 permutations. A permutation P value of less than 0.05 was considered to be statistically significant. The pairwise LD coefficients (r^2^) between SNPs were calculated to evaluate the structure of LD on 5q31-33 in 368 Thai malaria patients. The frequencies of haplotypes consisting of rs2069812, rs2299015, rs2299014, rs2243677, rs2522414, rs2299013, rs2252775, rs2522394, rs2245460, rs2301713, rs3798135, rs2237060, rs2074369, rs2240032, rs1881457, and rs1800925 were estimated only for this haplotype block. All the statistical analyses were performed by using the Haploview software version 4.0 [10]. The allelic state for each SNP (i.e., ancestral or derived) was inferred based on the genome sequence of *Pan troglodytes *(chimpanzee), obtained from the NCBI BLAST database (Table 1). When the genomic sequence of chimpanzee was not available, one of *Macaca mulatta *(rhesus macaque) was used.

## Results

### Association test

**Figure 1:**
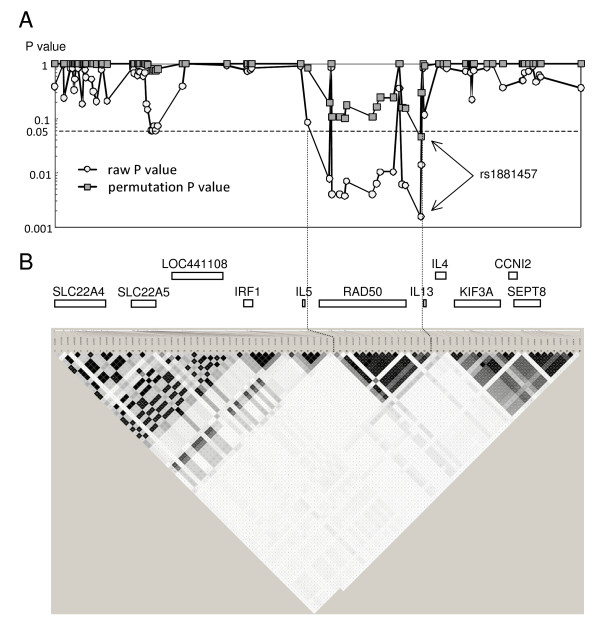
**Association P values and LD structure of 82 SNPs on 5q31**. Association P values and LD structure of 82 SNPs on 5q31. (A) The raw P value (open circle) and permutation P value (shaded square) for each SNP. (B) Pairwise LD measured by r2 between 82 SNPs. White, shades of grey, and black squares indicate no LD (r^2 ^= 0), intermediate LD (0 < r^2 ^< 1), and strong LD (r^2 ^= 1), respectively.

Eighty-two SNPs including rs1800925 were analysed to evaluate the association of the 5q31 region with severity of malaria (Table 1). The permutation P value as well as the raw P value was calculated for each SNP to avoid any false positive findings due to multiple testing (Table 1 and Figure 1A). Only rs1881457 showed a significant association with severe malaria (raw P value = 0.002 and permutation P value = 0.046) and no SNPs in the other genes showed any such association (permutation P value > 0.05). When a derived allele is focused on in association test, rs1881457-C may be referred to as a protective allele against severe malaria.

### LD structure

In previous study, rs1800925 was found to be associated with severe malaria [7]. Since rs1881457, showing the strongest association in the present study, is closely located to rs1800925, these SNPs may be in LD. In addition, a number of SNPs near rs1881457 and rs1800925 showed also raw P values of less than 0.05 (Table 1 and Figure 1), thus suggesting that some, if not all, of these SNPs are in the same haplotype block. To clarify the structure of the LD around rs1881457 and rs1800925, r^2 ^values between the 82 SNPs were calculated. The LD analysis for the 5q31 region revealed that all the SNPs showing low P values were in a distinct haplotype block spanning 97 kb from rs2069812 to rs2240032 (Figure 1B). This block contained the *RAD50 *gene and the promoter of *IL13*, but none of the other candidate genes such as *IL4*, *IL5*, and *IRF1*.

Six frequent haplotypes were observed in the detected block and two of which, haplotypes 1 and 4, bore rs1881457-C (Table 2). Both haplotypes showed a decreased frequency in severe malaria patients in comparison to those with mild malaria, thus suggesting that the association of rs1881457-C with the protection against severe malaria was not caused by a specific haplotype.

**Table 2:** Estimated haplotype frequencies in malaria patients.

**Haplotype^a^**	**Estimated frequency**	
	**Severe malaria**	**Mild malaria**

1: CCTGGGCATCAACTCT	0.095	0.163
2: TATACCAGATGATCAC	0.723	0.637
3: CAGACCAGATGATCAC	0.03	0.018
4: TATACCAGATGATCCC	0.025	0.04
5: CATACCAGATGATCAC	0.057	0.045
6: CAGACCAGATGCTCAC	0.038	0.048

^a^The haplotype consists of rs2069812, rs2299015, rs2299014, rs2243677, rs2522414, rs2299013, rs2252775, rs2522394, rs2245460, rs2301713, rs3798135, rs2237060, rs2074369, rs2240032, rs1881457, and rs1800925. Only haplotypes with the frequency of more than 0.02 either in severe malaria or in mild malaria were presented.

## Discussion

In this study rs1881457 was found to be significantly associated with severe malaria, and this SNP was included in a haplotype block encompassing the whole *RAD50 *gene and the promoter of *IL13 *(Figure 1). Together with MRE11 and NBS1, RAD50 forms a conserved multiprotein complex, MRE11-RAD50-NBS1 (MRN), which plays an important role in double-strand break repair, cell cycle checkpoint control, meiotic recombination, and telomere maintenance [11-13]. In the immune system, the MRN complex is involved in B cell-specific immunoglobulin gene diversification (e.g., Ig class-switch recombination, somatic hypermutation, and gene conversion) [14,15]. The polymorphisms of *RAD50 *may therefore influence the affinity and/or effector functions of antibodies. The *IL13 *gene encodes a immunoregulatory cytokine (Th2 cytokine) produced by activated Th2 cells. The Th2 cytokines down-regulate macrophage activity, and inhibit the production of pro-inflammatory cytokines such as TNF and IL1. It has been reported that increased concentrations of TNF and IL1β in serum are observed in severe malaria patients [16]. The *IL13 *promoter polymorphisms may influence the expression of *IL13*. Thus, both *RAD50 *and *IL13 *seem to be plausible candidate genes associated with severe malaria.

The genes encoding the Th2 cytokines *IL5*, *IL13*, and *IL4 *are subject to coordinate regulation and are expressed in a cell lineage-specific manner [17,18]. The expressions are regulated by a locus control region (LCR) located within a 25 kb region containing the 3' portion of *RAD50 *[19]. Interestingly, the LCR is included in the haplotype block associated with severe malaria, raising a possibility that polymorphisms which influence the LCR activity could account for the observed association with the severity of malaria.

Since rs1881457 is located in the promoter region of *IL13*, the nucleotide change at this site may affect the binding ability of some transcription factor. The TFSEARCH (TFSEARCH: Searching Transcription Factor Binding Sites, ) program based on the TRANSFAC databases [20] was used to examine the possibility. The result indicated no possible binding site of transcription factor at rs1881457 regardless of alleles (rs1881457-A and rs1881457-C) with the default setting (threshold score = 0.85). Therefore, rs1881457 itself may not be a primary polymorphism associated with severe malaria, even though rs1881457 showed the strongest association observed in this study.

Among *IL13 *polymorphisms, rs1800925 in the *IL13 *promoter has been reported to be associated with various diseases [21-24]. This SNP is located within a putative primate-specific cis-regulatory element [25] and has been shown to affect the promoter activity of *IL13 *[25] and IL13 production [26]. In the present study rs1800925 and rs1881457 had a high r^2 ^value (r^2 ^= 0.73). Therefore, the possibility that rs1800925 is a primary SNP and the significant association of rs1881457 with severe malaria resulted from LD between these SNPs is not excluded. The future functional and association studies for rs1881457, rs1800925, and other polymorphisms, including those not analyzed in the present study, may thus help us to better understand the genetic susceptibility to severe malaria.

## Conclusion

A haplotype block spanning 97 kb encompassing *RAD50 *gene and *IL13 *promoter region that was associated with severity of malaria was identified in a Thai population.

## Competing interests

The authors declare that they have no competing interests.

## Authors' contributions

NI carried out the genotyping, helped to conduct statistical analyses, and wrote the manuscript. NN and KT helped to genotype the samples. JP, PN and HH collected blood samples, extracted DNA, and helped to genotype the samples. JP participated in the design of the study and coordination. NT was involved in the interpretation of the data and preparation of the manuscript. JO conceived of the study, and participated in its design, performed statistical analyses, and helped to write the manuscript. All authors read and approved the final manuscript.
